# PP48 Cost-Effectiveness Of Mepolizumab In Children And Adolescents With Refractory Severe Eosinophilic Asthma In Brazil

**DOI:** 10.1017/S0266462324002198

**Published:** 2025-01-07

**Authors:** Ivan Ricardo Zimmermann, Flávia Tavares Elias, EricaTatiane da Silva

## Abstract

**Introduction:**

Asthma affects individuals of all ages and is the most common chronic disease among children. The Brazilian Unified Health System (SUS) reimburses the use of mepolizumab as an additional maintenance treatment for severe eosinophilic asthma in adults. This study aimed to evaluate the cost-effectiveness of expanding the reimbursement of mepolizumab to patients aged six years and older.

**Methods:**

The model included patients aged six years and older refractory to inhaled corticosteroid (ICS) plus long-acting beta-agonist (LABA) treatment. Baseline characteristics and outcomes were based on clinical trials and national hospitalization data. A Markov model with monthly cycles considered the health states without exacerbation, with exacerbation (including the need for oral steroids, emergency room admission, or hospitalization), and death. Direct medical costs (rate: USD1=BRL4.93) associated with all treatments were estimated from public health reimbursement sources. Quality-adjusted life years (QALY) was the primary outcome. Deterministic (Tornado) and probabilistic (Monte Carlo simulations) sensitivity analyses were conducted using Microsoft Excel.

**Results:**

Considering the current mepolizumab price (USD964.49) reimbursed by the Ministry of Health (MOH), the incremental cost-effectiveness ratio (ICER) was USD137,384.92/QALY (95% confidence interval: USD92,625.64, USD208,542.44), exceeding the USD24,333.86 threshold proposed by the National Committee for Health Technology Incorporation (Conitec). Using the average price from local public purchases (USD417.74), the ICER was USD96,673.51/QALY. Considering the past proposed discount from the manufacturer (USD390,93) and dose fractionation in children up to 12 years, the ICER was estimated in USD50,574.06/QALY. The most impactful parameters were the utility without exacerbation, mepolizumab cost, and relative reduction in exacerbation rate.

**Conclusions:**

Considering current reimbursement prices, mepolizumab use in children and adolescents with refractory severe eosinophilic asthma is not cost effective in Brazil. Alternative scenarios with price discounts and dose fractionation may alter these conclusions.

